# Data on the role of leadership in developing expertise in teaching in developing country

**DOI:** 10.1016/j.dib.2018.03.137

**Published:** 2018-04-03

**Authors:** Sayabek Ziyadin, Natalya Shash, Dina Kenzhebekova, Gulmira Yessenova, Ulan Tlemissov

**Affiliations:** a Al-Farabi Kazakh National University, Almaty, Kazakhstan; b Plekhanov Russian University of Economics, Moskva, Russia; c A. Baitursynov Kostanay State University, Kostanay, Kazakhstan; d JSC “Academy of Finance” of The Ministry of Finance of The Republic of Kazakhstan, Almaty, Kazakhstan; e International University of Kyrgyzstan, Bichkek, Kyrgyzstan

## Abstract

This article has researched role of leaders in developing expertise in teaching and their influence on teachers in secondary school in Kazakhstan. Also, how principles can affect to educators developing to meet needs and challenges of today's trends of teaching and learning. The following research report has been precisely written to evaluate the exact role of leadership practices in the development of expertise in teaching and in what manner the expert teachers or the principals help to develop expertise across various departments of the schools.

## Specifications table

**Table:** 

Subject area	*Education, management in education, economics,*
More specific subject area	*Role of leaders in developing expertise in teaching.*
Type of data	*Graph, chart*
How data was acquired	*Review of publications, journals and articles and even conduction of a number of surveys and interviews*
Data format	*Raw, analyzed*
Experimental factors	*90 teachers were surveyed to determine the influence of leadership in developing expertise in teaching*
Experimental features	*The role of leadership was determined*
Data source location	*Semey, Kazakhstan, 50.4233°N, 80.2508°E*
Data accessibility	*State if data is with this article or in public repository; if public repository, please explicitly name repository and data identification number, and provide a direct URL to data.*
Related research article	*The data are available with this article*

## Value of the data

• The data presented in this article is one of the first research where were determined the role of leadership in developing expertise in teaching in developing country such as Kazakhstan.
• The data could be used by other researchers for further deep research and as the data to analyse.
• The data could be compared to other researches.
• This data allows other researchers and readers to extend their viewpoint about the secondary school in Kazakhstan.

## 1 Data

The data of this article provides information about role principals and leaders in developing expertise in teaching in the secondary school in Kazakhstan.

## 2 Experimental design, materials and methods

### 2.1 The role of leadership in secondary school (Kazakhstan) settings in developing expertise in teaching

For the collection of data, a survey was conducted for the teachers so as to analyse their viewpoints and perceptions about the current and potential leadership activities. For surveying, a questionnaire is prepared with a number of questions and defined range of answers through which the respondents are asked to select their answers. The questionnaire medium is also adopted so that the participants could choose their answers without any pressure and hesitation and become more involved with the activities being carried out in the schools. Respondents were selected from thirty secondary schools in Semey area, whereby every school produced three participants: 90 respondents as teachers were chosen from each secondary school. The country been chosen here is the Kazakhstan. The respondents were reached via mails and other online platforms and in some cases personal interviews were also conducted. No permission was asked from the respective schools because the participants were called and informed about research at the individual level. To select participants were used the simple random sampling techniques in order to diminish biases and increase the trustworthiness of the research [2].

The following research report has been precisely written to evaluate the exact role of leadership practices in the development of expertise in teaching and in what manner the expert teachers or the principals help to develop expertise across various departments of the schools in Kazakhstan. The role of the teachers is quite pivotal in shaping the future of the students and this could be done at the initial level of school life only [1]. Hence, this report has been conducted to understand the elements of leadership and their influence on the teaching behaviour. A number of ideas have been collected over the chosen topic. In this regard, the necessity for change is present in every country and organisation in the marketable world. If an organisation does not change and innovate regarding market needs, or does not meet current challenges, then it will cease to operate or become increasingly non-competitive [3].

Below is an excerpt from the survey depicting the prime questions of the questionnaire.

Question 1. Are there any effective leaders across your school above the teachers?

• Yes
• No
• Have no idea

**Fig. 1:**
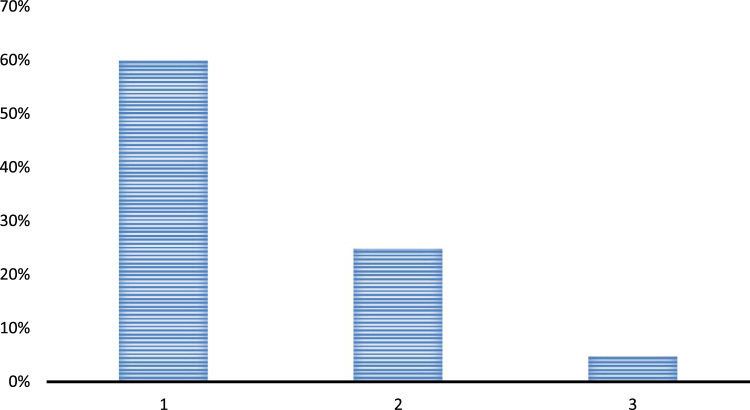
Presence of effective leaders in the schools 1. Yes, 2. No, 3. No idea.

**Fig. 2:**
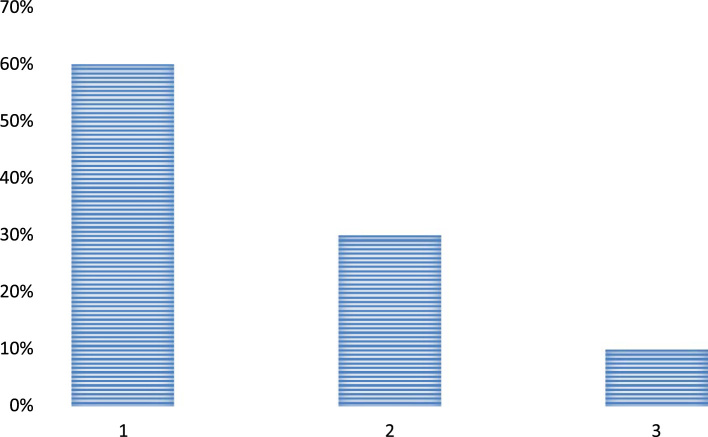
Role of principals in teachers' performance at the schools 1. Yes, 2. No, 3. Cannot be determined.

**Fig. 3:**
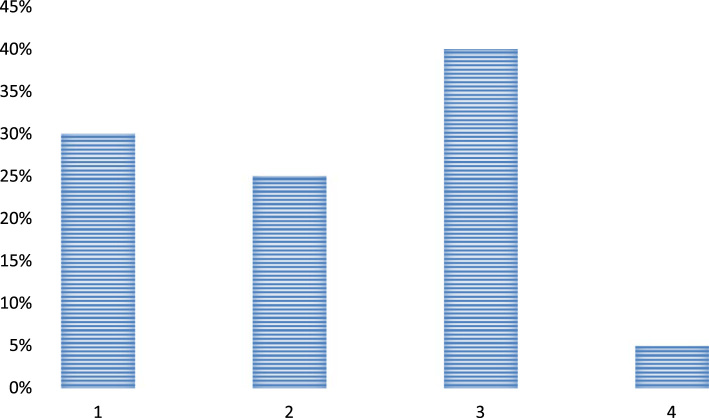
Prime qualities instilled in teachers by leaders 1. Diligence, 2. Great lecture delivery, 3. More passion towards job, 4. No qualities are instilled.

**Fig. 4:**
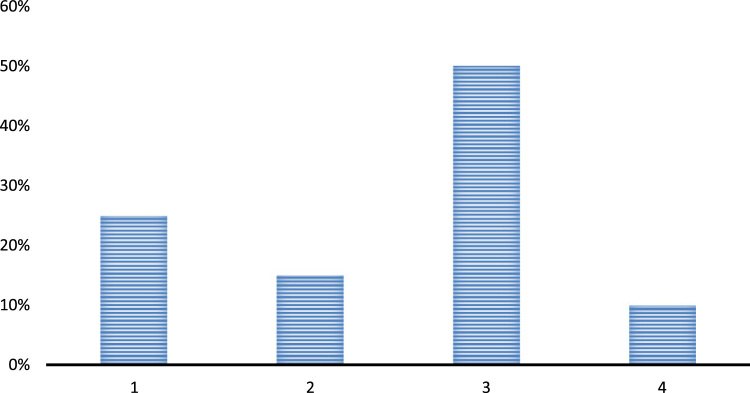
Purpose of CPD 1. Development of the personal qualities, 2. Professional development, 3. Both, 4. Haven’t heard of this concept before.

**Fig. 5:**
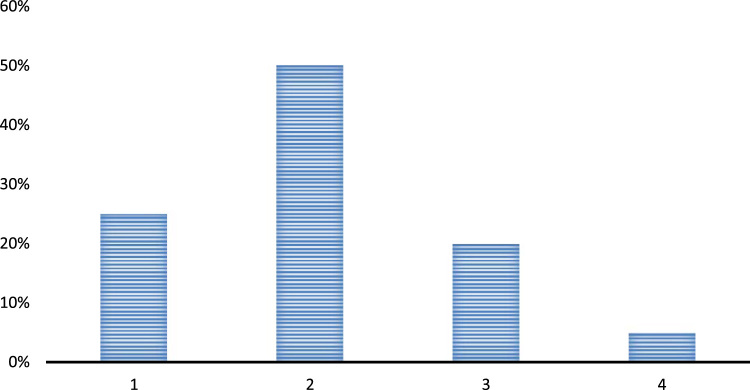
Presence of ASTs concepts at schools 1. Yes, but few, 2. Yes, many, 3. No, 4. Have no idea of this concept.

**Fig. 6:**
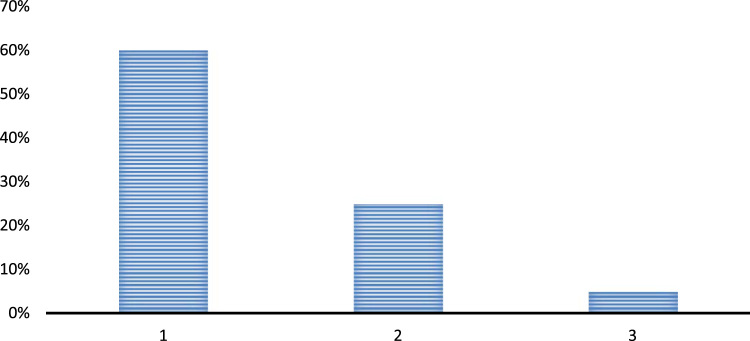
Influence of ASTs practices in improving performance of other teachers 1. Yes, 2. No, 3. Cannot be determined.

**Fig. 7:**
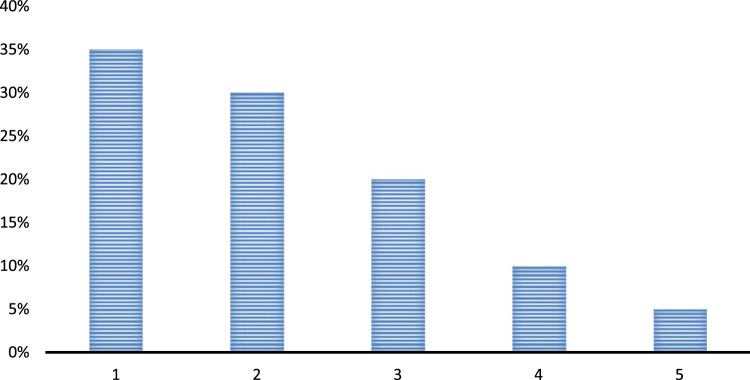
Leadership elements in improvement of performance of teachers 1. Strongly Agree, 2. Agree, 3. Neither agree nor disagree, 4. Disagree, 5. Strongly disagree.

The above chart depicts that most of the respondents agree to this fact that there are ample of leaders in their schools and most of the crucial decisions are being taken under the influence of these leaders. Moreover, the presence of leaders has allowed them to attain a different level of expertise in teaching and learning. Around 25% of the respondents still believe that there are no effective leaders in their schools and they are not getting a chance to work under a guidance of good leaders. Hence, their teaching abilities and talents are not being exploited in a proper manner (Fig.2). (Fig. 1, Fig. 2, Fig. 3, Fig. 4, Fig. 5, Fig. 6, Fig. 7

Question 2. Is there any substantial role of the principals in improving the teachers’ teaching and learning?

• Yes
• No
• Cannot be determined

The above analysis of the participants’ responses shows that the principals are playing a very important role in improving the performance of the teachers at the schools. Most of the respondents also consider that the presence of principals is quite crucial in determining the teaching abilities of the teachers. Some of the respondents still think that the teachers are themselves responsible for enhancement or degradation of their teaching and learning qualities and they are not influenced by the presence and absence of any leaders at their schools (Fig.2).

Question 3. What prime qualities a leader could instill in a teacher while working in a school?

• Diligence
• Great lecture delivery
• More passion towards job
• No qualities are instilled

The above chart depicts that the leaders are quite crucial in a school and tends to instill a number of qualities in the teachers. Most of the respondents consider that a good leader basically encourages a teacher to work hard in order to teach their pupils in a better manner. The respondents also equally consider that the teachers also learn to be more passionate towards the job by the leaders and they feel more encouraged at the workplace by maintaining a great balance between their job and personal life. Some of the participants in the survey also consider that a good leadership improves the lecture delivery style and quality of the teachers due to which the students are more and more involved in their studies and other classroom activities (Fig.3).

Question 4. For what purpose is the Continuous Professional Development (CPD) is required in teaching and learning?

• For development of the personal qualities
• Professional development
• Both
• Haven’t heard of this concept before

The above chart suggests that most of the respondents are aware of the concept of Continuous Professional Development and even considers that the CPD elements have a potential to influence a teacher both at the professional and personal levels. With the introduction of this concept a teacher easily excels in his professional life and by being satisfactory with his work he simply manages his personal issues very well. So both the lives are highly influenced by the concepts of CPD. Also, some of the respondents haven’t heard of this concept prior to this survey because the leadership elements have not been so strong at their schools till now (Fig.4).

Question 5. Are there any Advanced Skills Teachers (ASTs) at your school?

• Yes, but few
• Yes, many
• No
• Have no idea of this concept

The above chart shows that in most of the secondary schools in the Kazakhstan the concept of ASTs exists and is being acknowledged by all the participants. The Advanced Skills Teachers are not working for their individual development, but they are promoting the weaker teachers to learn and perform in an outstanding manner. Due to this concept many teachers have managed to become a great asset for their institution. The above chart indicates that most of the respondents have replied that many ASTs elements exists in their schools and are being readily practiced by all the participants of the school. Only few of the respondents believe that there is no such concept in their school and the teachers cannot be good leaders in a way guiding the co-workers in terms of performance improvements (Fig.5).

Question 6. Does the existence of ASTs influence the teaching abilities of the other teachers in the school?

• Yes
• No
• Cannot be determined

From the above graph, it could be easily deduced that the presence of Advanced Skill teachers is quite important for a school as they not only work for their individual benefits but also encourage the other teachers to deliver good lectures and improve their skills in their subjects. Among the respondents, some of them still think that the concept of ASTs is not acceptable in their schools and only principals could work as a leader by encouraging all the teachers in a successful direction. According to their perceptions the teachers could not learn to expertise in their works from their co-workers (Fig.6).

Question 7. The leadership elements in a school improve the performance of the teachers. To what extent do you agree to this statement?

• Strongly Agree
• Agree
• Neither Agree nor Disagree
• Disagree
• Strongly Disagree

The above chart depicts that the leadership elements are quite crucial for a school and teachers learn a lot from the leaders. The influence of good leaders is always positive, and they guide the teachers to perform in a best manner. Moreover, the teachers learn to tackle any tough situation in a composed manner when they are working under any good leader. Some of the respondents still think that the leaders are not crucial in any school and the role of the principal is just restricted to framing and passing rules and policies (Fig.7).

In conclusion, firstly, despite some negative roles of leaders, they are main figures and driving force in educational organisation to improve quality (balance between job and personal live, developing expertise in teaching) of teachers and teaching. Secondly, Continuous Professional Development is main instrument that can meet the modern challenges and trends of a volatile world as teachers have the opportunity to develop themselves constantly.
